# Mapping the landscape of dissemination and implementation science across the CTSA consortium: A multi-domain environmental website scan

**DOI:** 10.1017/cts.2026.10760

**Published:** 2026-05-29

**Authors:** Jing Li, Naomi Duffort, Salisa Westrick, Renee Heffron, Abigail Gamble, Bertha Hidalgo, Katherine Mills, Kirsten Dorans, Kristine Ria Hearld, Larry R. Hearld, Lisa Zubkoff, Peter T. Katzmarzyk, Michael Mugavero

**Affiliations:** 1 The University of Alabama at Birmingham, Birmingham, AL, USA; 2 Auburn University Harrison College of Pharmacy, Auburn, AL, USA; 3 The University of Mississippi Medical Center, Jackson, MS, USA; 4 Tulane University School of Public Health and Tropical Medicine, New Orleans, LA, USA; 5 Pennington Biomedical Research Center, Baton Rouge, LA, USA

**Keywords:** dissemination science, implementation science, translational science, capacity building

## Abstract

Despite substantial investment in clinical and translational research, only a small proportion of evidence-based interventions are adopted and sustained in routine practice, contributing to persistent delays between discovery and population benefit. Dissemination and implementation (D&I) science is a critical discipline for addressing this gap, and the NIH Clinical and Translational Science Award (CTSA) program (established 2006) has been strategically positioned as a national infrastructure to advance D&I capacity. We conducted a national environmental scan of publicly available websites and documents from all 66 CTSA hubs (May–July 2025), using a structured extraction tool to capture D&I-specific activities across seven domains: institution and community partnerships, formal D&I organizational structures within the CTSA, consultation services, collaborative programming, training opportunities, educational offerings, and pilot funding mechanisms. Findings reveal substantial heterogeneity in D&I science activities across CTSA hubs; 45% had a formal D&I unit, 54% offered D&I consultation services, and 37% provided collaborative programming. Structured workforce development was limited: 12% offered D&I-focused training grants, 15% offered structured educational programs, and 15% provided D&I-specific pilot funding. Consultation models varied widely in scope, access, and evaluation practices. These findings demonstrate uneven development of D&I science infrastructure across CTSAs and highlight opportunities to strengthen capacity nationally.

## Introduction

It has long been recognized that the publication of trial results and clinical guidelines, while necessary, are often insufficient to ensure the widespread adoption and sustained use of evidence-informed interventions and programs in routine clinical and public health settings. Over three decades ago, Lomas observed, “for a handful of findings that are so compelling, passive diffusion works and you don’t need the rest of the dissemination and implementation (D&I). But there aren’t many penicillins left in the world.”[1] Moreover, the “17-year odyssey” from scientific discovery to routine delivery in healthcare settings – achieved by only a small proportion of evidence-based interventions (∼20%) – remains a major barrier to optimizing individual and population health outcomes [2]. In response, the field of D&I science has gained increasing attention over recent decades as disciplines dedicated to understanding and developing strategies that facilitate the integration of evidence-based practices into clinical, community, and public health settings, promote their long-term sustainability, support their long-term use, and disseminate their use to new settings.

The National Institutes of Health (NIH) has played a prominent role in building capacity for D&I science. Supporting research to understand and overcome the barriers impeding the translation of research findings into routine practice was identified as a priority of the NIH Roadmap Initiative in 2007 [3]. Other initiatives articulated the core values guiding the NIH’s investment in D&I science – namely, rigor and relevance, efficiency and speed, collaboration, capacity building, and learning from cumulative knowledge in other disciplines [4]. Established in 2006, the NIH Clinical and Translational Science Award (CTSA) program has provided a robust national infrastructure to advance D&I science through a network of over 60 academic institutions and their academic, community, and public health partnerships. Chartered in 2016, a cross-CTSA dissemination, implementation, and knowledge translation workgroup put forth recommendations to situate D&I across the translational research setting. Through the *Integrative Framework of Dissemination, Implementation and Translation (IFDIT)*, this working group illustrated the critical role of D&I science principles spanning the entire translational science spectrum, from basic research to population health [5]. In 2021, a cross-domain working group of the CTSA consortium, comprising experts in methods and processes, workforce development, evaluation, stakeholder engagement, and D&I, issued recommendations for integrating D&I science principles within CTSA award programs [6]. These recommendations include embedding D&I science perspectives in CTSA activities; establishing dedicated D&I structures (e.g., cores); intentionally incorporating D&I science principles, skills, and knowledge into workforce development programs (e.g., NIH T-and K-training programs); and fostering clinical, public health, and community partnerships, the ultimate adopters and implementers of evidence-based interventions and programs.

These recommendations align with the National Center for Advancing Clinical and Translational Science (NCATS) vision, to “bring more treatments for all people more quickly,” with D&I science playing a central role in the NCATS 2025–2030 strategic plan [7]. As the CTSA program evolves [8], the integration of D&I science principles holds promise for strengthening strategic management, training and outreach, resource allocation and pilot programs, clinical and translational science research programs, and collaborative partnerships, advancing the NCATS vision.

Notably, the CTSA’s cross-domain working group underscored the importance of longitudinal evaluation of D&I science programs and resources across CTSAs [6]. The CTSA Dissemination, Implementation, and Knowledge Translation Working Group conducted a survey of CTSA leaders (55.2% response rate). This 2017 CTSA survey showed wide variation in D&I support across hubs, with only about half reporting direct funding for D&I programs (54.1%), training (51.4%), or projects (59.5%) and many relying on indirect support such as CTSA promotion without dedicated funding [9]. Given the rapid evolution of D&I science, CTSA program requirements, and national expectations for translation over the past several years, an updated environmental scan is needed to assess current D&I resources and capacity, identify progress and persistent gaps, and inform more coordinated and equitable strategies across CTSA hubs.

## Materials and methods

The authors developed a standardized data extraction tool to systematically capture D&I science resources and programs offered by CTSA hubs. The tool included a yes/no checklist assessing the presence of offerings across seven domains: (1) hub interinstitutional partnerships, (2) a dedicated D&I science core or section, (3) D&I consultation services, (4) collaborative D&I science activities, (5) D&I training grants and career development programs, (6) formal D&I educational offerings, and (7) D&I pilot grant funding. For domains coded as present, additional information was collected to characterize the scope of each offering. From May to July 2025, a single reviewer (ND) abstracted data from publicly available websites and documents for 66 CTSA hubs. Specifically, for each CTSA hub, the study systematically examined the CTSA hub website and relevant affiliated pages (e.g., training programs, community engagement units). Examples of publicly available documents included annual reports, consultation request forms, pilot funding announcements, training program descriptions, webinar materials, and other programmatic summaries posted on CTSA or institutional websites. When available, we also reviewed linked resources such as newsletters and evaluation reports. To enhance data accuracy, the study incorporated two additional approaches. First, members of the study team leveraged professional connections across CTSA hubs to verify and, where possible, supplement information captured through the environmental scan. Second, we conducted targeted outreach to four CTSA hubs to confirm the accuracy of the extracted information and obtain clarifications when needed. Descriptive statistics were used to summarize the frequency and percentage of hubs offering specific D&I resources, supplemented by qualitative notes on program characteristics and context. A second reviewer (JL) independently reviewed a random sample of five hub websites to validate the extracted data.

## Results

Findings from the environmental scan of CTSA hubs (*n* = 66) publicly available websites and published documents are provided by the seven domains prioritized by the data extraction tool. Table 1 provides additional information for the presence of D&I core/sections and the D&I domains across CTSA Hubs.

**Table 1.** CTSA hub dissemination and implementation (D&I) science characteristics determined by an environmental scan of publicly available websites and published documents for CTSA hubs (*N* = 66)

UL1 (*N* = 25)		*N*/%		
D&I science core		Formal D&I core (8/32%)	Limited D&I service with a formal structure (2/8%)	No visible D&I presence (15/60%)
		Formal and limited (10/40%)		
Consultation services	8/32%	8/80%		0/0%
Collaborative activities	6/24%	5/50%		1/7%
Training grants/career development programs	2/8%	2/20%		0/0%
Formal educational offerings (e.g., certificate, degree)	4/16%	4/40%		0/0%
Pilot funding	4/16%	3/30%		1/7%
UM1 (*N* = 41)				
D&I science core		Formal D&I core (22/54%)	Limited D&I service with a formal structure (6/15%)	No visible D&I presence (13/32%)
		Formal and limited (28/68%)		
Consultation services	27/66%	27/96%		0/0%
Collaborative activities	18/44%	16/57%		2/15%
Training grants/career development programs	6/15%	6/21%		0/0%
Formal educational offerings (e.g., certificate, degree)	6/15%	6/21%		0/0%
Pilot funding	6/15%	6/21%		0/0%
Total (*N* = 66)				
D&I science core		Formal D&I core (30/45%)	Limited D&I service with a formal structure (8/12%)	No visible D&I presence (28/42%)
		Formal and limited (38/58%)		
Consultation services	35/53%	35/92%		0/0%
Collaborative activities	24/37%	21/55%		3/11%
Training grants/career development Programs	8/12%	8/21%		0/0%
Formal educational offerings (e.g., certificate, degree)	10/15%	10/26%		0/0%
Pilot funding	10/15%	9/24%		1/4%

Overall, there were notable differences between UL1 (*N* = 25) and UM1 (*N* = 41) hubs. In general, UM1 hubs demonstrated greater visibility of D&I activities, including D&I cores (68% vs. 40%), consultation services (66% vs. 32%), collaborative programming (44% vs. 24%), and training grants (15% vs. 8%).

### Inter-institutional partnerships

A majority of CTSA hubs (39, 59%) engaged 1 to 4 academic or clinical hub partners (range [0,13]), while 8 hubs (12%) had 5 or more partners, with some functioning as central coordinating centers for broader regional networks. On websites, description of partnerships ranged from structured lists to vague references, which limits comparability across hubs.

### Presence of a distinct D&I science core/section

Across the 66 CTSA hubs, there was considerable heterogeneity in the organization and structure of distinct D&I science units. Forty-five percent (*n*=30) have a formal D&I core, section, or dedicated program, whereas 12% (*n*=8) appear to provide some D&I services without a formal structure, and the remaining 42% (*n* = 28) show no discernible DIS activities on their publicly facing websites (Table 1).

Hubs with formal D&I structures are primarily located at large academic medical centers and often include faculty who are national thought leaders in D&I science. At other hubs, D&I activities are embedded within existing units (e.g., community engagement or evaluation) or are not clearly integrated into the CTSA hub infrastructure. D&I resources are frequently difficult to identify or poorly publicized on institutional websites, limiting accessibility. Support for D&I structures typically draws on a mix of institutional funds and grant support from NCATS and other sponsors, with CTSA hub D&I programs often leveraging broader institutional initiatives and expertise.

### D&I consultation services

Over half of the hubs (*n* = 35, 53%) offer D&I consultation services (Table 1). The remaining 47% either do not offer services or have no publicly listed information available. Notably, all hubs offering consultation services had either a formal or limited D&I structure within their CTSA.

A commonality across D&I consultation services is the range of consultation topics offered, which frequently include study design and methods, selection of D&I frameworks, theories, and models, grant proposal development and review, identification of collaborators or community partners, and selection of implementation strategies and outcomes. However, considerable variation exists across institutions in their organization, delivery models, fee structures, and program evaluation practices. Service models range from brief, drop-in office hours to multi-step consultations incorporating structured intake and follow-up processes. Intake procedures differ as well – some institutions employ detailed systems requiring requestors to complete intake forms specifying their needs and expected timelines, whereas others use a more informal approach, providing only a contact person’s email. Many programs include a triage process to route requests based on consultant expertise and availability, with response times varying from two business days to two weeks. Eligibility also differs, with most services available to faculty, some extended to postdoctoral fellows and students, and access for external collaborators often unspecified. Consultation formats are often not clearly defined; when described, they include in-person or virtual delivery and one-on-one or group-based approaches. Fee structures vary widely – many programs offer free consultations for internal investigators, though some provide only the first few hours (e.g., 2 to 4 hours) at no cost before charging hourly rates or applying tiered fees for extended or specialized services such as communications design, media production, or long-term project management. Finally, few publicly report data on the uptake or effectiveness of D&I consultation services such as satisfaction and the number of grant submissions tied to consultations. Collectively, these variations highlight the diversity and evolving nature of D&I consultation service models across institutions.

### Collaborative D&I science activities

Collaborative events – such as seminar series, annual symposia, working groups, and grand rounds – are among the most visible D&I capacity-building activities highlighted on CTSA hub websites, with approximately 37% (*n* = 24) offering such programming (Table 1). These events generally fall into four categories. First, many institutions host recurring seminar series or workshops, typically offered monthly or quarterly, that cover topics ranging from research methodologies and specific content areas (e.g., mental health, maternal health) to strategies for sustaining implementation efforts. These sessions are most often delivered virtually, with some hubs alternating between virtual and in-person formats to enhance accessibility. Second, annual symposia – often half-or full-day events – feature national speakers and allow for in-depth engagement with emerging issues in D&I science. Third, informal working or interest groups function as ongoing communities of practice that foster networking, collaboration, and shared problem-solving. Finally, some hubs offer grand rounds or forum-style sessions focused on healthcare quality, translational research, or methodological innovations relevant to D&I research.

Notably, no single CTSA hub hosts programming across all four categories, highlighting a clear opportunity for strengthening capacity-building efforts through a more comprehensive portfolio of collaborative events.

### D&I science training grants and career development programs

Although nearly all CTSA hubs reference NIH-and NCATS-supported training mechanisms (e.g., TL1, KL2, T32, and K12), only eight hubs (12%) offer programs that explicitly include structured D&I training (Table 1). These D&I-focused programs most often use dedicated K12 or T32 mechanisms that combine didactic coursework, mentored research projects, and enrichment activities to build advanced competencies in implementation research.

Across hubs, existing D&I training opportunities share several defining features. All programs concentrate on D&I science within healthcare settings, reflecting the field’s strong clinical orientation. With the exception of one program, they are disease agnostic, preparing trainees to apply implementation principles across a variety of clinical conditions and service contexts. Program duration ranges from one to two years, providing trainees with sustained, structured support to develop and conduct implementation research.

### Formal D&I educational offerings

The availability of formal D&I educational programs and continuing learning opportunities varies widely across CTSA hubs. Common offerings include journal clubs, short courses, and lunchtime webinars; however, only 15% (*n* = 10) of hubs (Table 1) provide tiered learning pathways or sustained programs designed to build D&I science competencies over time. Structured programming spans a broad range of intensity, from multi-course graduate certificate programs in D&I to standalone semester-long courses, short courses or summer workshops, and brief 1–2 hour introductory sessions. Many of these offerings are framed as research professional development rather than formal training programs. Few hubs offer continuing education credits or certificates, and most programs are oriented toward early-stage investigators or clinicians with emerging interests in D&I science. Consequently, opportunities remain limited for mid-career and established investigators – many of whom apply D&I principles in their work but have not had access to structured, formal training in the field.

### D&I pilot grant funding

Pilot grant programs are ubiquitous across CTSA hubs, but only 10 hubs (15%) explicitly offer D&I-focused pilot proposal opportunities (Table 1). Available funding typically ranges from $10,000 to $50,000 for one-year awards. These may be managed by the CTSA itself or through aligned centers (e.g., public health institutes, community engagement programs). These pilot grants are typically disease agnostic and focus on implementation science projects within clinical and community settings. A few pilots, however, fund policy-related projects and those that involve adapting study designs and measures.

## Discussion

We report findings from a national environmental scan of publicly available websites and documents from all 66 CTSA hubs to characterize current D&I science programs and resources across the network. Substantial heterogeneity was observed in the scope and structure of D&I science programming across hubs. These findings are consistent with a 2017 survey of CTSA leaders, in which 54.1% of respondents reported direct support for D&I science programs and resources, while also offering greater granularity on specific D&I science offerings aligned with recent recommendations from a cross-domain CTSA working group [6,9].

Our findings contribute to a body of literature focusing upon the roles of CTSA hubs in building D&I science capacity and infrastructure. For example, the UCLA-led Southern California CTSA hub engaged academic, public health, and delivery system stakeholders to identify needs and develop recommendations for enhancing D&I science through multisectoral regional partnerships [10]. Key priorities included D&I science education and training, consultation and technical assistance, development of tools and resources, and fostering synergies across hub functions. While hubs like UCLA have been at the forefront of building D&I capacity, our environmental scan shows that many hubs have yet to establish dedicated D&I science programs and resources. Commonly reported barriers include limited funding, insufficient D&I-trained workforce, and gaps in understanding of D&I science, consistent with findings from a recent CTSA leader survey [9].

Our findings build upon a growing portfolio of literature published by CTSA working groups related to situating D&I science across the translational science spectrum and recommending opportunities for growth. In the context of CTSAs, the IFDIT identifies and delineates the vital role of D&I science principles at each stage from basic research to population health [5]. The intentional integration of D&I science principles along the translational science spectrum is foundational to the research conducted within each of the stages (i.e., T0–T4), as well as the research application between stages, toward accelerating the delivery of scientific discoveries to the individuals and populations who would derive benefit. Beyond theoretical guidance, a cross-domain CTSA working group has issued recommendations to realize the full potential of D&I science, including establishing dedicated D&I structures (currently present in 45% of CTSAs), developing the workforce through targeted education and training programs, and fostering strategic partnerships. Our study indicates that relatively few hubs offer formal courses, certificate or degree programs (15%), or D&I-focused T-and K-level training grants (12%), highlighting substantial opportunities to expand and diversify postdoctoral pathways. Similarly, reporting of clinical, public health, and community partnerships remains limited, reflecting an area for further development and visibility.

We highlight the prominence of D&I science consultation services, provided by over half of CTSA hubs (53%). Recently, a group from the University of California San Diego evaluated the core functions and forms of their D&I science consultations, reporting that a majority of requests related to grant proposals (54%), followed by ongoing projects (25%) [11]. Topics most discussed included implementation science principles (55%), guidance centered on D&I science methods (47%), and study/project design. The manuscript further described the alignment of the consultation guidance with the level of D&I science competencies and tracked the outcomes of consultations. As CTSAs continue to grow D&I science consultative capacity, this manuscript points to a potential opportunity to monitor and evaluate consultations across the network in terms of content, competency level, and outcomes, to facilitate cross-CTSA evaluation.

The observed heterogeneity in D&I infrastructure across CTSA hubs has important implications beyond the CTSA program itself. Variation in the availability of D&I resources, such as consultation services, training opportunities, and pilot funding, may directly influence investigators’ ability to design, implement, and sustain evidence-based interventions. Hubs with more developed D&I capacity are better positioned to support research that is implementation-ready, whereas limited infrastructure may contribute to persistent gaps between evidence generation and real-world uptake. As a result, uneven D&I capacity across CTSAs may translate into variability in the pace, quality, and consistency of translational research outputs across institutions.

Given their role as a national research infrastructure, CTSAs are uniquely positioned to advance D&I science and accelerate the translation of evidence into practice. Strengthening D&I capacity across hubs could include standardizing core resources, expanding workforce development pathways, and increasing access to consultation and pilot funding. In addition, greater cross-hub collaboration and sharing of best practices may help reduce duplication of effort and promote more consistent adoption of effective models. By leveraging their networked structure, CTSAs can serve as a catalyst for disseminating D&I knowledge and fostering scalable, sustainable approaches to implementation, ultimately enhancing the impact of clinical and translational research on population health.

These findings should be interpreted in light of several study limitations. Our assessment of D&I science resources and programs was limited to publicly available websites and published materials, so it is possible that offerings across the seven domains exist but were not captured if not documented online.

In conclusion, this environmental scan provides a national snapshot of D&I science programs and resources across all 66 CTSA hubs, spanning seven key domains. Our findings build on the growing literature on D&I capacity within CTSAs, highlighting that while most hubs offer structured programs and consultations, offerings of formal educational courses, workforce development and training grants, and dedicated D&I-focused pilot grant programs remain limited. Aligned with recent recommendations from a cross-domain CTSA working group, these results point to opportunities to (1) standardize reporting of D&I cores, services, and training programs, (2) expand structured workforce development programs (e.g., T32 and K12), (3) increase D&I-focused pilot grant programs, and (4) embed and standardize evaluation metrics across consultation and other D&I programs offered by CTSAs.
